# Neofunctionalization of a Noncoding Portion of a DNA Transposon in the Coding Region of the Chimerical Sex-Determining Gene *dm-W* in *Xenopus* Frogs

**DOI:** 10.1093/molbev/msac138

**Published:** 2022-06-28

**Authors:** Shun Hayashi, Kosuke Suda, Fuga Fujimura, Makoto Fujikawa, Kei Tamura, Daisuke Tsukamoto, Ben J Evans, Nobuhiko Takamatsu, Michihiko Ito

**Affiliations:** Department of Bioscience, School of Science, Kitasato University, 1-15-1 Kitasato, Minamiku Sagamihara, Kanagawa 252-0373, Japan; Department of Bioscience, School of Science, Kitasato University, 1-15-1 Kitasato, Minamiku Sagamihara, Kanagawa 252-0373, Japan; Department of Bioscience, School of Science, Kitasato University, 1-15-1 Kitasato, Minamiku Sagamihara, Kanagawa 252-0373, Japan; Department of Bioscience, School of Science, Kitasato University, 1-15-1 Kitasato, Minamiku Sagamihara, Kanagawa 252-0373, Japan; Department of Bioscience, School of Science, Kitasato University, 1-15-1 Kitasato, Minamiku Sagamihara, Kanagawa 252-0373, Japan; Department of Bioscience, School of Science, Kitasato University, 1-15-1 Kitasato, Minamiku Sagamihara, Kanagawa 252-0373, Japan; Department of Biology, McMaster University, Life Sciences Room 328, 1280 Main Street West, Hamilton, ON, Canada L8S 4K1; Department of Bioscience, School of Science, Kitasato University, 1-15-1 Kitasato, Minamiku Sagamihara, Kanagawa 252-0373, Japan; Department of Bioscience, School of Science, Kitasato University, 1-15-1 Kitasato, Minamiku Sagamihara, Kanagawa 252-0373, Japan

**Keywords:** sex determination, transposon, chimeric gene, interspecific hybridization, frog, transcription factor

## Abstract

Most vertebrate sex-determining genes (SDGs) emerge as neofunctionalized genes through duplication and/or mutation of ancestral genes that are involved with sexual differentiation. We previously demonstrated *dm-W* to be the SDG in the African clawed frog *Xenopus laevis* and found that a portion of this gene emerged from the masculinization gene *dmrt1* after allotetraploidization by interspecific hybridization between two ancestral species around 17–18 Ma. *dm-W* has four exons consisting of a noncoding exon 1, *dmrt1*-derived exons 2 and 3, and an orphan exon 4 (Ex4) of unknown origin that includes coding sequence (CDS). In this study, we searched for the origin of Ex4 and investigated the function of the CDS of this exon. We found that the Ex4-CDS is derived from a noncoding portion of the *hAT-10* family of DNA transposon. Evolutionary analysis of transposons and determination of the Ex4 sequences from three other species indicated that Ex4 was generated before the diversification of most or all extant allotetraploid species in subgenus *Xenopus*, during which time we hypothesize that transposase activity of this *hAT* superfamily was active. Using DNA–protein binding and transfection assays, we further demonstrate that the Ex4-encoded amino acid sequence increases the DNA-binding ability and transrepression activity of DM-W. These findings suggest that the conversion of the noncoding transposon sequence to the CDS of *dm-W* contributed to neofunctionalization of a new chimeric SDG in the ancestor of the allotetraploid *Xenopus* species, offering new insights into de novo origin and functional evolution of chimerical genes.

## Introduction

In vertebrates, sex-determining genes (SDGs) evolved independently many times (Ito and Mawaribuchi 2013; Pan et al. 2016). Most SDGs emerged via neofunctionalization (Mawaribuchi et al. 2012); for example, mammalian *Sry* (*Sex-determining region of Y*) evolved through allelic divergence from *Sox3* (*Sry-box 3*) in the ancestor of therian mammals (Foster and Graves 1994), and *dmy/dmrt1bY* and *dm-W* independently evolved from duplicates or partial duplicates of *dmrt1* (*doublesex and mab-3 related transcription factor 1*) in ancestors of the teleost fish medaka and the frogs of the subgenus *Xenopus*, respectively (Matsuda et al. 2002; Nanda et al. 2002; Yoshimoto et al. 2008; Bewick et al. 2011). *Sox3* and *dmrt1* encode transcription factors with two different DNA-binding domains—HMG (high mobility group) and DM domains, respectively. The SDGs *dmy*, *dm-W*, and *Sry* each have higher substitution rates than their respective paralogous or gametologous genes, *dmrt1*, *dmrt1*, and *Sox3* (Mawaribuchi et al. 2012). *Dmrt1*, which probably originated in the most recent common ancestor of vertebrates (Mawaribuchi et al. 2019), functions in testis formation to promote somatic-cell masculinization gene and germ cell development (Smith et al. 2009; Matson et al. 2010, 2011; Yoshimoto et al. 2010; Masuyama et al. 2012; Zarkower 2013; Zhao et al. 2015; Fujitani et al. 2016; Mawaribuchi, Musashijima, et al. 2017; Ge et al. 2018).

Early during evolution of the subgenus *Xenopus*, an allopolyploidization event between two closely related diploid species generated the tetraploid ancestor of all species with 36 chromosomes; subsequent allopolyploidization events generated octoploid (72 chromosome) and dodecaploid (108 chromosomes) species (reviewed in Evans 2008). The initial allopolyploidization event in subgenus *Xenopus* is estimated to have occurred around 17–18 Ma (million years ago) and resulted in two “subgenomes”—L and S—that are respectively derived from each diploid ancestor (Session et al. 2016) (see supplementary fig. S1, Supplementary Material online). Although it resides on chromosome 2 of the L subgenome, the SDG *dm-W* evolved from the S subgenome homeolog of *dmrt1* (*dmrt1.S*) and is carried by several species in subgenus *Xenopus* including highly diverged species pairs such as *Xenopus clivii* and *X. laevis* (Bewick et al. 2011; Cauret et al. 2020). The contrast between the genealogical relationship (closer to *dmrt1.S*) and its genomic context (in subgenome L) coupled with its phylogenetic distribution in many species in subgenus *Xenopus* argues that this gene became established in its current location after allotetraploidization but before the diversification of extant allotetraploids in subgenus *Xenopus* (Bewick et al. 2011; Mawaribuchi, Takahashi, et al. 2017; Cauret et al. 2020; see supplementary fig. S1, Supplementary Material online). Analysis of sequence homology suggests that *dm-W* is a chimeric gene with Ex2 and Ex3, which encode the DM domain being derived from *dmrt1.S* and Ex1 and Ex4 being of unknown origin (Yoshimoto et al. 2008; Mawaribuchi, Takahashi, et al. 2017). Thus, *dm-W* has no homology to *dmrt1* exons 4–6, which encodes a transregulation domain (Yoshimoto et al. 2010). Moreover, it remains unclear whether the distinct components of *dm-W* (Ex1, Ex2 + 3, and Ex4) became juxtaposed before allopolyploidization in the diploid ancestor of subgenome S and then later tandemly translocated to subgenome L after allotetraploidization, or whether juxtaposition of these components occurred after translocation of Ex2 + Ex3 to subgenome L in the allotetraploid ancestor of subgenus *Xenopus*.

In *X. laevis*, *dm-W*-expression or *dm-W*-knockdown in ZZ or ZW tadpoles induces ovarian and testicular formation, respectively (Yoshimoto et al. 2008, 2010). The sequence of the DNA-binding domain of DMRT1 is highly conserved across mammals, amphibians, and fish (Murphy et al. 2007; Yoshimoto et al. 2010; Ogita et al. 2020). In vitro assays indicate that DM-W antagonizes the transcriptional activation of downstream genes by DMRT1 (Yoshimoto et al. 2010). This is consistent with a model for sex determination in *X. laevis* wherein DMRT1 activates masculinizing genes in males by binding to their cis-elements in ZZ primordial gonads, whereas DM-W represses the transcriptional activity of DMRT1 in females by competing with DMRT1 for binding to cis-regulatory elements in ZW primordial gonads, leading to feminization (Yoshimoto et al. 2010; Yoshimoto and Ito 2011). In vitro assays indicate that the DNA-binding affinity to a consensus binding sequence is higher for DM-W than DMRT1 (Ogita et al. 2020), which is consistent with this model. These findings suggest that *dm-W* functions as a dominant-negative protein that counteracts the masculinization factor *dmrt1*, and that DM-W is a repressor of primary male sexual differentiation in *X. laevis* (Yoshimoto and Ito 2011).

Transposable elements (TEs) are able to change genomic locations or generate new copies in of themselves, and are generally considered to not benefit the host genome (i.e., “selfish DNA”). However, TEs may also influence genome architecture, gene expression, and gene content in beneficial ways (Anderson and Springer 2018; Ågren and Clark 2018; Bourque et al. 2018). For example, the generations of at least two SDGs might be linked to TE activity that influence transcription: TE-cis-regulatory modules influence expression of the SDG *gsdf* on the Y chromosome of sablefish, and in the fighting fish, *dmrt1* on the X chromosome is subject to TE-induced epigenetic silencing (Herpin et al. 2021; Wang et al. 2022). Several eukaryotic protein-coding genes originated (partially) from TEs, such as *Gary* (which arose from a transposase genes) or *Rag* (which arose from retrotransposons and DNA transposons) (Fugmann 2010; Alzohairy et al. 2013). TEs are also the source of various long and functionally important noncoding RNAs (reviewed in Bourque et al. 2018). However, of ∼900 genes that arose de novo in an ancestor of seven closely related *Caenorhabditis* species including *C. elegans*, <1% arose from TEs (Zhang et al. 2019), and this mode of gene origin appears to also be infrequent in insects (Wissler et al. 2013). Not surprisingly, most examples of novel CDSs that arose from TEs involve coding sequence (CDS) of TEs, as opposed to from non-CDS. There are very few examples of noncoding portions of TEs becoming exon(s), resulting in the generation of different isoform proteins. Two examples include a retrotransposon-derived non-CDSs that evolved to function through alternative splicing as a CDS in bovine EP3 receptor gene and a novel TE-derived exon in mouse *Sry* (Shimamura et al. 1998; Miyawaki et al. 2020).

As discussed above, *dm-W* is a chimerical SDG that arose in an ancestor of allotetraploid *Xenopus* frogs that triggers female sexual differentiation in some *Xenopus* species; one portion of this gene arose from partial duplication of the masculinizing gene *dmrt1* but the other portion is of unknown origin. The goals of this study are to determine where the orphan exon 4 of *dm-W* came from and what its function may be. To this end, we first deployed a bioinformatic strategy to search for sequences homologous to Ex4 and found that it was derived from a noncoding region of the DNA transposon *hAT-10* family belonging to widespread eukaryotic *hAT* (*hobo*, *Ac*, and *Tam-3*) superfamily. We then performed in vitro and cotransfection assays that demonstrate that the Ex4-derived amino acid sequences both increase the DNA-binding ability of DM-W and the transrepression activity of DM-W on DMRT1. These findings indicate that the evolutionary origin and neofunctionalization of *dm-W* was achieved by a combination of partial gene duplication of *dmrt1* coupled with the recruitment of a non-CDS transposon sequence into a functionally important component of the CDS of *dm-W*.

## Results

### *dm-W* Ex2 and Ex3 and Flanking Regions are Derived from *dmrt1.S*

To clarify the origin of the components of the chimeric gene *dm-W*, we first compared the genomic sequences of *X. laevis dm-W*, *dmrt1.S*, *dmrt1.L*, and *X. tropicalis dmrt1*, which were obtained from the *X. laevis* genome v9.2 and *X. tropicalis* genome v10.0 (http://www.xenbase.org/entry/). Although *X. laevis* has an allotetraploid genome consisting of L and S subgenomes, *X. tropicalis* has a diploid genome whose ancestor diverged from the ancestor of subgenus *Xenopus* before *dm-W* originated (Bewick et al. 2011; Session et al. 2016). Approximately 80 kbp nucleotide sequences in and around the four genes were aligned, and homologous regions were estimated using mVISTA (supplementary fig. S2, Supplementary material online). We found that not only Ex2 and Ex3, but also small portions of the 3′ region of intron 1, intron 2, and the 5′ region of intron 3 of *dm-W* shared sequence homology with the corresponding regions of the three *dmrt1* genes. In contrast, Ex1, the 3′ region of intron 3, Ex4, and 5′/3′-flanking sequences of Ex1 and Ex4 have no identifiable homology with any of these three *dmrt1* genomic sequences (supplementary fig. S2, Supplementary material online). These results indicate that the genomic region from the 3′ region of intron 1 to the 5′ region of intron 3 of the ancestral *dmrt1.S* (which resides on chromosome 1S) was duplicated into one of the two ancestral chromosome 2L. In addition, we estimated a phylogenetic tree of these genes using the homologous sequences of the *dm-W*’s introns 1–3 in addition to Ex2 and Ex3. We confirmed that *dm-W* is more closely to *dmrt1.S*, not *dmrt1.L*, with higher confidence values (supplementary fig. S3, Supplementary material online) than those reported previously (Bewick et al. 2011; Mawaribuchi, Takahashi, et al. 2017).

### The CDS of *dm-W* Ex4 Evolved from a Non-CDS of the *hAT-10* DNA Transposon Family

**Fig. 1.**
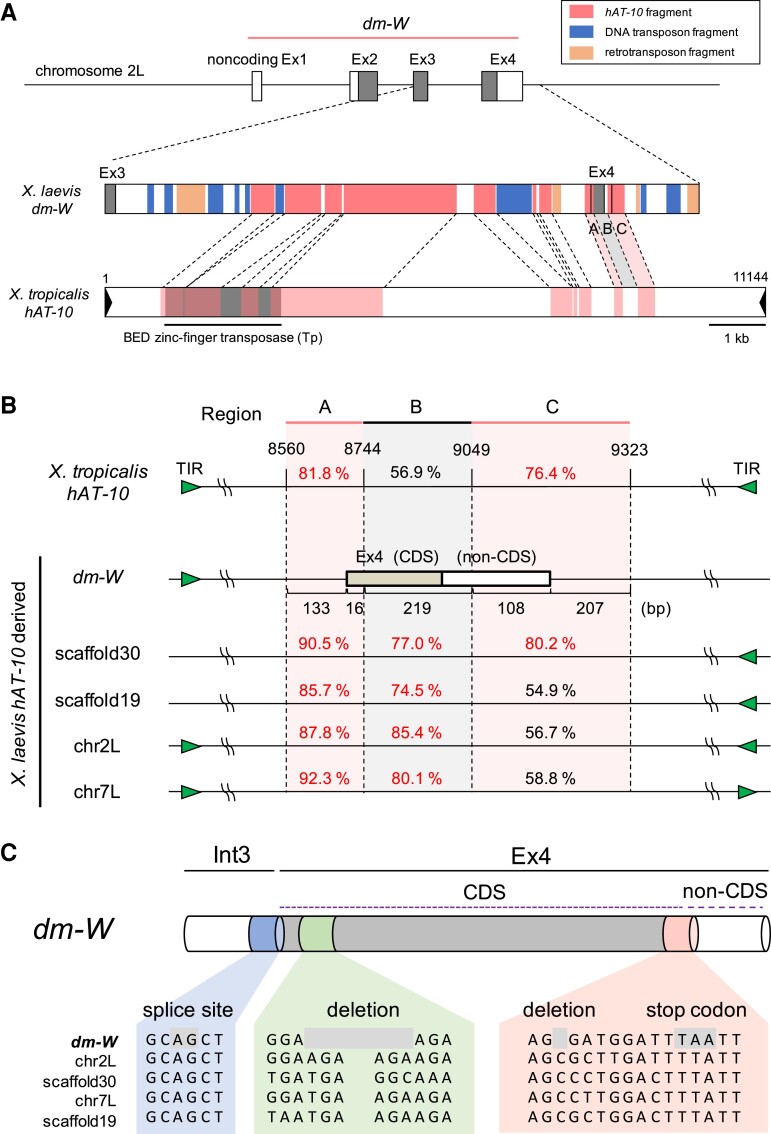
*hAT-10* DNA transposon-derived fragments on the *dm-W* intron 3 and exon 4 (Ex4). (*A*) Distributions of transposon-derived DNA fragments in and around Ex4 of *Xenopus laevis* (*Xl*) *dm-W* and comparison to *X. tropicalis* (*Xt*) *hAT-10* DNA transposons. Noncoding and coding portions of exons of *dm-W* are indicated with white and gray boxes, respectively (upper). Colored boxes in *X. laevis dm-W* represent TE distribution by CENSOR as indicated (middle). A box in *X. tropicalis hAT-10* DNA transposon shows homologous regions to the *dm-W hAT-10*-derived ones (lower). (*B*) Schematic comparison among the *dm-W* Ex4 and its corresponding regions from *Xt hAT-10* to *Xl hAT-10*-derived sequences. The *hAT-10*-derived sequences were classified into three regions (named A, B, and C) based on sequence similarity. Region A (133 bp of the third intron and 16 bp of Ex4 in *dm-W*) and C (108 bp of Ex4 and 207 bp downstream) shared high sequence identity among them, whereas region B (219 bp of the Ex4) has lower sequence identity between *Xt hAT-10* and each *Xl hAT-10* transposon-derived sequence. Nucleotide sequence identity (%) with *dm-W* is shown on each region. Noncoding and coding exons are represented by white and gray boxes, respectively. The green triangle shows a terminal inverted repeat (TIR). (*C*) A partial comparison of nucleotide sequences within and adjacent to the Ex4-CDS among the five *hAT-10* transposon-derived sequences from *X. laevis* in (*B*). Splicing acceptor site AG, a deletion, and a sequence for a stop codon are shaded.

We previously reported the accumulation of TEs in W-specific regions of *X. laevis* (Mawaribuchi, Takahashi, et al. 2017). We examined the TE density in 80 kb region in and around *dm-W* using the CENSOR program (www.girinst.org/censor/index.php) (Kohany et al. 2006). There is a higher proportion of TEs in the *dm-W*-containing region (69.1%) than in the *dmrt1.L* or *dmrt1.S*-containing region (34.0% or 27.9%, respectively) as indicated by the red–blue gradient in supplementary fig. S4, Supplementary Material online. Interestingly, TEs were identified not only within introns 1–3, but also within Ex4 of *dm-W* (supplementary fig. S5, Supplementary material online). We then performed a focused search for TE-like sequences in and around Ex4. Here *hAT-10* DNA transposon-like fragments were found to comprise more than half of intron 3, a portion of the 5′ region of Ex4, and about 250 bp of the 3′-flanking region of Ex4 (fig. 1*A* and supplementary fig. S5, Supplementary material online). In Ex4, we identified three regions designated as A, B, and C spanning a total of 683 bp with sequence similarity to *X. tropicalis hAT-10*: region A (133 bp of the 3′ region on intron 3 and 16 bp of the 5′ region on Ex4), region B (219 bp of the central portion of Ex4), and region C (108 bp of the 3′ portion of Ex4 and 207 bp of the 3′-flanking region downstream of Ex4) (fig. 1 and supplementary fig. S6*A*, Supplementary material online). The A and C regions were recognized by CENSOR, and had relatively high nucleotide identity (81.8% and 76.4%, respectively) with the corresponding sequences of *X. tropicalis hAT-10*, whereas the region B between A and C had a lower identity (56.9%) with *X. tropicalis hAT-10* (fig. 1*B*). We then searched for similar nucleotide sequences to these regions containing the CDS of *dm-W* Ex4 in *the X. laevis* genome using BLAST, and found four sequences on scaffolds 19, 30, chromosome 2L, and chromosome 7L. All included *hAT* transposon fragments that had higher sequence similarities with the regions A and B of *dm-W* (85.7–92.3% and 74.5–85.4%, respectively) than with *X. tropicalis hAT-10* (fig. 1*B* and supplementary table S1, Supplementary material online). Using the CENSOR program, we also detected 14 bp of terminal inverted repeats, which are also a feature of DNA transposons, around *dm-W* Ex4 and the four *hAT-10*-like sequences (supplementary figs. S6*A* and *B*, Supplementary material online). Together, these results indicate that these five sequences with high similarity in the *X. laevis* genome—including *dm-W* Ex4—are derived from the *hAT-10* family. The *hAT-10* transposon family contains one protein gene encoding a BED zinc-finger transposase consisting of 1033 amino acids (Hellsten et al. 2010). We found the corresponding region to this *hAT-10* CDS in intron 3 of *dm-W*, but not in Ex4 of *dm-W* (fig. 1*A*). Therefore, we conclude that the CDS of *dm-W* Ex4 evolved from a non-CDS of a *hAT-10* transposon to now encode the C-terminal region of the DM-W protein.

To further clarify how the transposon-derived non-CDS evolved into CDS of Ex4, we performed sequence alignment among the sequences around the Ex4-coding region and its corresponding sequences from the four Ex4-like sequences derived from *hAT-10* using MUSCLE (fig. 1*C* and supplementary fig. S6*A* and *B*, Supplementary material online). The comparison identified two point mutations consisting of a deletion and substitution that eliminated a frame-shift mutation and formed a stop codon in the ancestral *hAT-10*-derived sequences, thereby resulting in the generation of a 71 amino acids CDS in the C-terminal of *dm-W* that are encoded by Ex4 (fig. 1*C*).

### The Time of Allotetraploidization in Subgenus *Xenopus* Coincides with a High Rate of Replicative Transposition of *dm-W* Ex4-like *hAT* Transposons

**Fig. 2.**
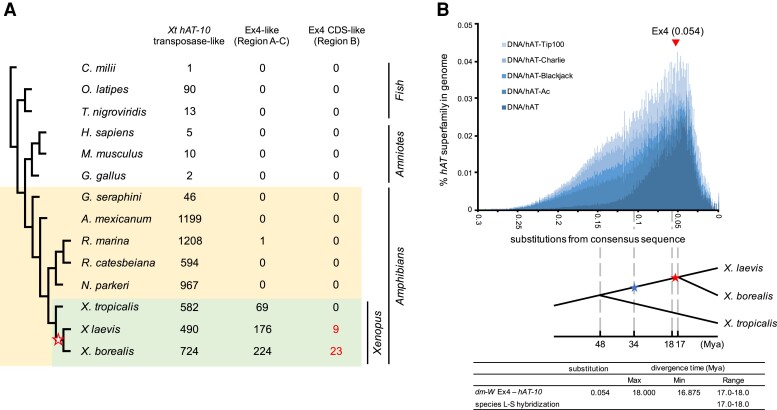
Molecular evolution of *hAT* superfamily, *hAT-10* family, and *hAT-10*-derived Ex4 in *Xenopus* frogs. (*A*) Copy numbers of *Xt hAT-10* transposase (Tp)-like sequences, *hAT-10-*derived Ex4-like sequences (A–C in fig. 1*B*), *hAT-10-*derived Ex4-CDS-like sequences [B in A] in 14 vertebrate species, including anuran amphibians (*Xenopus laevis*, *X. borealis*, *X. tropicalis*, *Nanorana parkeri*, *Rana catesbeiana*, and *Rhinella marina*), an urodele amphibian (*Ambystoma mexicanum*), a caecilian amphibian (*Geotrypetes seraphini*), a bird (*Gallus gallus*), mammals (*Homo sapiens* and *Mus musculus*), teleost fish (*Oryzias latipes* and *Tetraodon nigroviridis*), and a cartilaginous fish (*Callorhinchus milii*). GenBank assembly accession of 11 species except for the three *Xenopus* species used is shown in supplementary fig. S3, Supplementary Material online. (*B*) A repeat landscape of *hAT* superfamily consisting of the five families, as inferred in the *X. laevis* genome using RepeatMasker (upper). The *y*-axis and *x*-axis show percentages of each family on the genome and Jukes-Cantor-corrected divergence, respectively. The estimated divergence time of the *hAT-10*-derived regions on and around *dm-W* Ex4 from the *hAT-10* consensus sequence is shown by a triangle on the landscape. After the ancestors of *X. laevis* and *X. tropicalis* diverged at 48 Ma, speciation and hybridization of the predicted L and S species occurred at 34 and 17–18 Ma, respectively (Session et al. 2016).

To clarify the timing of divergence of *hAT-10* transposons from *dm-W* Ex4-related sequences, we blasted the *hAT-10* sequence in 14 vertebrate genomes, including eight amphibian species (fig. 2*A*). All the vertebrate genomes examined had at least one copy of a *X. tropicalis* (*Xt*) *hAT-10* transposase-like sequence. The caecilian and six nonamphibian species contained far fewer copies, including host–transposase fusion genes (Cosby et al. 2021), which might be derived from the *hAT* superfamily. However, all the amphibians except for the caecilian have hundreds of copies in their genomes. This suggests that *hAT-10* transposons expanded in urodeles and anurans and/or their most recent common ancestor after divergence from caecilians. *hAT-10-*derived Ex4-like sequences corresponding to regions A–C were found only in anuran amphibians and appeared to actively expand only in the *Xenopus* genus (fig. 2*A*). Notably, 9 and 23 copies of the Ex4-CDS-like sequence corresponding to region B were detected in the two allotetraploid species *X. laevis* and *X. borealis,* respectively, whereas no copies were found in the diploid species *X. tropicalis*. As well, ∼2–3 times as many sequences similar to regions A and C were identified in *X. laevis* and *X. borealis* compared with *X. tropicalis* (fig. 2*A*). Moreover, in the other allotetraploid *X. borealis*, we found six sequences of the *hAT-10*-derived transposon fragments sharing some sequence similarities with the three regions, A, B, and C of *dm-W* (80.1–89.7%, 71.4–76.3%, and 74.5–84.0%, respectively) (supplementary table S2 and fig. S7, Supplementary Material online), suggesting the existence of the ancestral *hAT-10* transposon with the Ex4-like sequence in the most common ancestor of *X. laevis* and *X. borealis*. Overall, this indicates that the rate of replicative transposition of this class of *hAT* transposons increased in African clawed frogs, and particularly so after divergence of the most common ancestor of *X. laevis* + *X. borealis* from the ancestor of *X. tropicalis*.

To understand evolutionary trends of *hAT* transposon superfamily in *Xenopus*, we constructed repeat landscape of the *hAT* superfamily consisting of *hAT-Tp100*, *hAT-Charlie*, *hAT-Blackjack*, *hAT-Ac*, and *hAT*, using *X. laevis* genome database and RepeatMasker (fig. 2*B*). The activation peak of the *hAT* superfamily was observed around 0.05 substitutions per site. The nucleotide substitution rate in *Xenopus* was estimated to be 3.0 × 10^−9^ to 3.2 × 10^−9^ substitutions per year (Session et al. 2016). Using this rate, we estimated the divergence time of the *hAT-10*-derived sequence in *dm-W* by comparing the sequence recognized as *hAT-10* by CENSOR and its corresponding one from a consensus *X. laevis hAT-10.* We calculated a median divergence to consensus of 0.054, which corresponds to an estimated peak transposon activity as 17.0–18.0 Ma. Interestingly, we recently found the activation peak of total DNA transposons in the two allotetraploid *Xenopus* species, *X. laevis* and *X. borealis* to be about 17.0 Ma, which is around the hybridization (Suda et al. 2022). These results suggest that the *dm-W*-ancestral *hAT-10* transposon was inserted into the ancestral chromosome 2L roughly around the time of allotetraploidization during the active peak period of DNA transposons including *hAT* superfamily, although we cannot definitively determine whether this was before or after this event.

### Emergence and Molecular Evolution of the Chimeric Gene *dm-W* Before Diversification of Most Species in Subgenus *Xenopus*

**Fig. 3.**
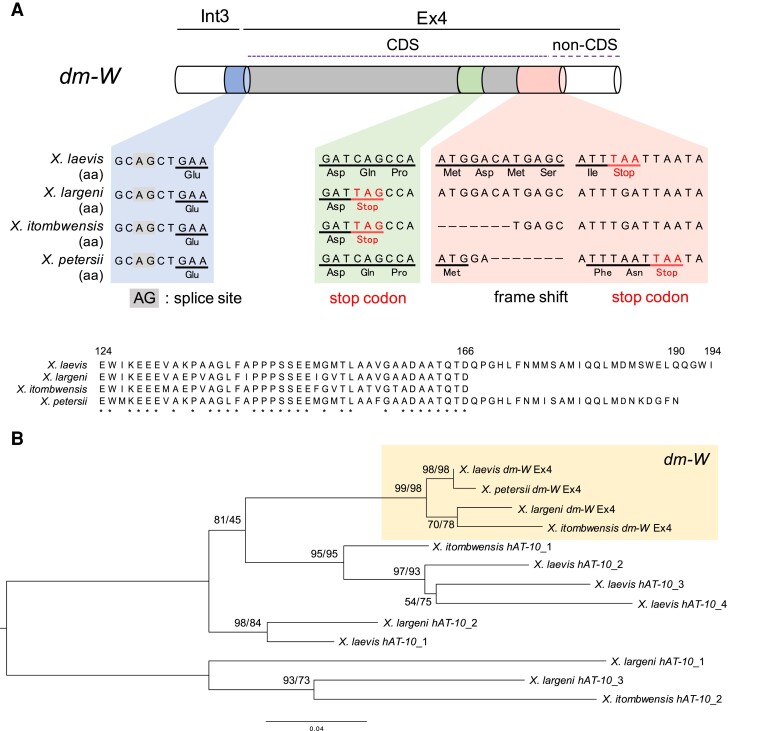
The nucleotide and deduced amino acid sequence alignments of the Ex4-CDS of *dm-W* among four allopolyploid *Xenopus* species. (*A*) A multiple nucleotide sequence alignment within and adjacent to Ex4-CDS of *X. laevis*, *X. largeni* (MCZ-A cryogenic 333), *X. itombwensis* (MCZ-A A136197), and *X. petersii*. Red font highlights the positions of TAG and TAA stop codons. (*B*) A multiple alignment of the Ex4-encoded amino acid sequences from the four DNA sequences in (*A*) (upper) and the ML phylogenetic tree of the Ex4 sequences and/or their corresponding *hAT-10*-derived sequences from *X. laevis*, *X. largeni*, *X. itombwensis*, and *X. petersii* (lower). Numbers at each node denote the ML/NJ bootstrap percentages of 1000 replicates.

To further understand the timing of emergence of Ex4, we tried to identify *dm-W* Ex4-CDSs from three allotetraploid species, *X. borealis*, *X. largeni*, and *X. petersii*, and one allooctoploid species, *X. itombwensis*. We performed PCR using genomic DNA and primer pairs designed by the conserved sequences of *X. laevis dm-W* and the four *hAT-10*-like sequences (supplementary fig. S6, Supplementary material online). Ex4-like sequences were obtained from *X. largeni*, *X. petersii*, and *X. itombwensis*, but not from *X. borealis.* Multiple sequence alignment and phylogenetic analysis revealed that the sequences obtained from the three species contained the Ex4-CDSs of *dm-W* (fig. 3*A*). The Ex4-CDSs in the three species were highly conserved with that of *X. laevis* and had two of the same mutations consisting of one nucleotide deletion and substitution as those of *X. laevis* (figs. 1*C* and 3*A*), suggesting that *dm-W* Ex4 in these species are homologous. Interestingly, the Ex4-CDSs from *X. itombwensis* and *X. largeni* or *X. petersii* encoded 43 or 67 amino acids, whereas *X. laevis* contained 71 amino acids (fig. 3*B*). These differences are caused by a single nucleotide mutation that generated a UAG stop codon in the upstream region of an ancestor of *X. itombwensis* and *X. largeni* and a frameshift mutation caused by the deletion of 16 nucleotides in the ancestor of *X. petersii* (fig. 3*A*). Exons 2 and 3 of *dm-W* were present in the ancestor of all species in subgenus *Xenopus* (Cauret et al. 2020). These results demonstrate that *dm-W* Ex4 was also present at least as early as the most recent common ancestor of *X. largeni*, *X. itombwensis*, *X. laevis*, and *X. petersii*—this ancestor diversified after divergence from the *muelleri* species group (*X. clivii*, *X. borealis*, *X. muelleri*, *X. fischbergi*, and *X. fraseri*) (Evans et al. 2015, 2019).

### The 71 Amino Acid Sequence Encoded by the *hAT-10* Transposon-derived Ex4 Increases the DNA-binding Affinity of DM-W

**Fig. 4.**
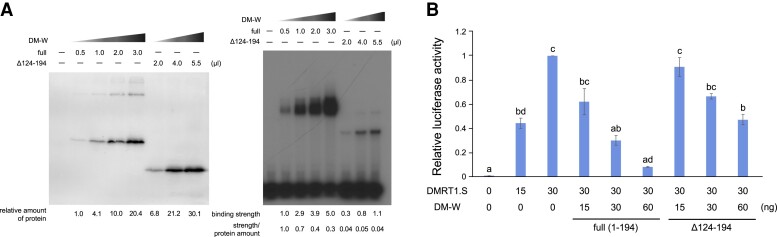
Effects of the transposon-derived Ex4 coding region in in vitro DNA binding and transrepression activities of DM-W. (*A*) In vitro DNA binding of DM-W (full length) and its C-terminal truncated protein, DM-W (Δ124–194), to the DMRT1-binding sequence. Flag-DM-W(full) and Flag-DM-W(Δ124–194) were produced by in vitro transcription–translation system and analyzed by Western blot analysis with an anti-FLAG antibody followed by an HRP-conjugated antimouse antibody (left). The relative intensity values were shown below. EMSA was performed using in vitro synthesized Flag-DM-W(full) (0.5, 1.0, 2.0, or 3.0 μl) or Flag-DM-W(Δ124–194) (2.0, 4.0, or 5.5 μl) and ^32^*P*-labeled double-stranded oligonucleotides containing the DMRT1-binding sequence. The relative intensity values to the protein amounts, binding strength, and ratio of strength/protein amount were shown below. (*B*) The luciferase reporter assay for the DM-W transrepression activity on DMRT1-driven transcription. The DMRT1-driven luciferase reporter and an expression plasmid for DMRT1.S and DM-W(full) or DM-W(Δ124–194) were transfected into HEK293*T* cells and posttransfection (24 h) luciferase activity was measured. Letters (a–d) indicate significant differences based on a one-way ANOVA, followed by the Tukey–Kramer HSD test (*P* < 0.01).

As detailed above, the *hAT-10-*derived Ex4 encodes the C-terminal region of DM-W, which consists of 71 amino acid residues in *X. laevis*. To clarify the function of this portion of the DM-W peptide, we examined the effect of the region on the DNA-binding ability of the DM domain encoded by Ex2 and Ex3. An in vitro DNA–protein binding assay by Electrophoretic Mobility Shift Assay (EMSA) using 30 bp of DM-W/DMRT1-binding DNA sequence (Yoshimoto et al. 2010) was performed for full-length DM-W and its C-terminal truncated protein DM-W (Δ124–194) carrying no Ex4-encoding 71 amino acid sequence (fig. 4*A*, left). The amount of each protein, which was produced by in vitro transcription/translation, was quantified using western blot analysis (fig. 4*A*, right). The assay showed that the full-length DM-W had a stronger binding ability to its binding sequence than the truncated DM-W peptide (Δ124–194) (fig. 4*A*). These results indicate that the Ex4-encoding 71 amino acid sequence increases the DNA-binding affinity of DM-W to a DM-W/DMRT1-binding DNA sequence, which is consistent with previous assays (Yoshimoto et al. 2010).

We next examined the effects of full-length and C-terminal truncated DM-W proteins on the transcriptional activity of DMRT1 using a luciferase reporter plasmid carrying four copies of the DM-W/DMRT1-binding cis-element, which were used for the in vitro DNA–protein binding assay. HEK 293 T-cells were transiently cotransfected with the reporter plasmid and expression plasmids for DMRT1.S, DM-W (full length), and/or truncated DM-W (Δ124–194). The reporter assay showed that DMRT1-driven transactivation activity was more repressed by DM-W (full length) than the truncated DM-W (Δ124–194) (fig. 4*B*). In other words, the *hAT-10* transposon-derived C-terminal region increases the transrepression activity of DMRT1.S by DM-W, compared with a truncated version of DM-W that lacks the *hAT-10* transposon-derived C-terminal region. Collectively, these findings indicate that the *hAT-10* transposon-derived Ex4 may enhance the DNA-binding ability of DM-W to DM-W/DMRT1-binding cis-elements.

## Discussion

Only about ten vertebrate SDGs have been identified so far; these encode various types of proteins, including transcription factors, a sex-steroid-synthesizing enzyme, and TGF-β signaling-related ligands and receptors (Mawaribuchi et al. 2012; Nagahama et al. 2021). Most SDGs were generated independently through duplication or allelic mutations in ancestral genes that functioned in gonadal differentiation. As discussed above, *Xenopus dm-W* and medaka fish *dmy* independently evolved from a broadly conserved, male-related transcription factor *dmrt1* into a male repressor and male inducer, respectively (Yoshimoto and Ito 2011). Parallel amino acid substitutions enhanced DNA-binding activities in ancestral genes of both *dm-W* and *dmy*, which suggested a common mechanism for the establishment of these SDGs (Ogita et al. 2020). In this study, we demonstrate that a non-CDS derived from a *hAT-10* DNA transposon evolved into the Ex4-CDS in the C-terminal region of DM-W, and that this addition strengthened the DNA-binding activity of the DM domain encoded by Ex2 and Ex3 (figs. 1, 2, and 4). These findings suggest that the molecular evolution from the non-CDS to the CDS was a fundamental event in the emergence of *dm-W* as a male repressor SDG.

*dm-W* is a chimeric gene (Yoshimoto et al. 2008), which is an unusual mode of origin among known SDGs. Although most therian orthologs of *Sry* have a single exon, mouse *Sry* encode two isoforms, single exon-derived SRY-S, and two exon-derived SRY-T (Miyawaki et al. 2020). The Ex2 gene of *Sry-T* encodes 15 amino acid sequence derived from a retrotransposon-derived sequence (a long interspersed nuclear element L3), which includes a degron that regulates protein degradation rates (Koren et al. 2018; Miyawaki et al. 2020). The participation of the L3 retrotransposon-derived sequence is hypothesized to maintain *Sry* function during rodent diversification (Miyawaki et al. 2020). Similarly, the *hAT-10* transposon-derived sequence of *dm-W* appears to influence the DNA-binding affinity of this protein, and is thus also of functional significance. However, there is a key difference between these SDGs in terms of their maintenance and emergence: the degron-CDS of rodent *Sry-T* might be responsible for the continued functioning of its sex determination’s role in some rodents even though this sequence is not required in other therian mammals. In contrast, the *hAT-10* transposon-derived sequence may have been essential for the ancestral neofunctionalization of *dm-W* into an SDG.

We previously proposed an evolutionary model for the relationship between SDGs and sex chromosomes, in which undifferentiated (homomorphic) sex chromosomes more frequently undergo sex chromosome turnover via the origin of new SDGs than do differentiated (heteromorphic) sex chromosomes (Mawaribuchi et al. 2012). *Xenopus laevis* has morphologically homomorphic sex chromosomes (Tymowska 1991). Recently, we demonstrated from sequence analysis of the genome that W and Z sex chromosomes, which are gametologous versions of chromosomes 2L, have ∼280 kb W-specific sequences containing *dm-W* and an ∼80 kb Z-specific sequence (Session et al. 2016; Mawaribuchi, Musashijima, et al. 2017). We also found that the W- and Z-specific regions have three (including *dm-W*) and one gametolog-specific genes, respectively, although apart from *dm-W* we do not know their function (Mawaribuchi, Musashijima, et al. 2017). In this study, we performed a detailed analysis of the molecular evolution of *dm-W* (fig. 2), indicating that *dm-W* arose around the time of allotetraploidization in subgenus *Xenopus*. It is possible that *dm-W* evolved as a new SDG in a newly evolved allotetraploid ancestor with an unstable sex-determining system after hybridization between two ancestral diploid species, both of which had homomorphic sex chromosomes. After the establishment of *dm-W* as SDG, homologous recombination suppression may have led to the accumulation of mutations and TEs.

Although most TEs are often recognized as “junk DNA” in host organisms, some TEs have important functions as long and small noncoding RNAs, and influence gene expression (Ariel and Manavella 2021). TEs can also participate in the formation of new protein-coding genes. For example, retrotransposon-derived elements play a role in signal transduction and mammalian evolution (Shimamura et al. 1998; Kaneko-Ishino and Ishino 2015). A recent comparative genomics approach identified ∼100 fusion genes with DNA transposon-derived transposase-CDSs, many of which might have evolved under functional constraints (Cosby et al. 2021). However, to our knowledge, no examples are known where DNA transposon-derived non-CDSs evolved into CDSs. In *dm-W*, it is possible that the conversion of a noncoding to coding region consisting of 40–70 amino acids was favored by natural selection because this sequence increased the DNA-binding affinity of DM-W.

**Fig. 5.**
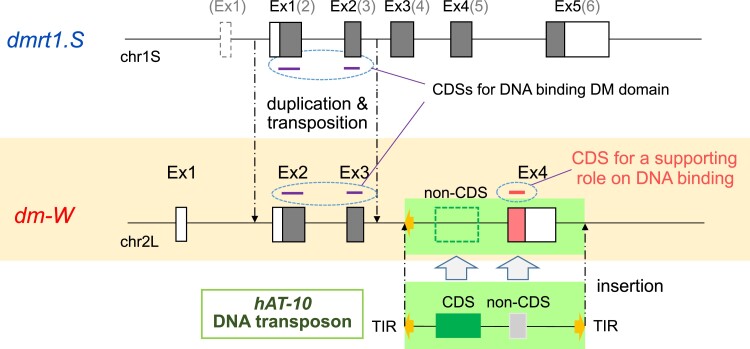
Proposed model for the emergence of the chimeric gene *dm-W* as SDG. The two closely related *Xenopus* species having two distinct genomes named as L and S were hybridized about 17–18 Ma (Session et al. 2016). After the interspecific hybridization, at least three independent insertion events into chromosome 2L led to the establishment of *dm-W* as a female SDG. The three events lead to the generations of noncoding Ex1 from promoter/enhancer for expression in gonadal somatic cells during sex determination, Ex2–Ex3 from the duplication of the S subgenome-derived ancestral *dmrt1.S*, and Ex4 from a *hAT-10* DNA transposon. A single nucleotide deletion and substitution in the non-CDS of the *hAT-10* transposon-derived sequence resulted in the Ex4-CDS, which resulted in a C-terminal region that strengthens the DNA-binding ability of the DM-W protein.

Based on these findings, we propose further investigation to elucidate the sequence of mutation events preceding the emergence and molecular evolution of SDG *dm-W* (fig. 5). The mixture of two subgenomes L and S by interspecific hybridization and allotetraploidization between two closely related *Xenopus* species about 17–18 Ma (Session et al. 2016) may have destabilized the existing two sex-determination systems that were present in the ancestral frog species, which also may have had homomorphic sex chromosomes. In some populations, the sex ratio could have been biased, which could have favored the establishment of *dm-W* as the sole SDG. In the case of *dm-W*, the most parsimonious scenario is that at least three independent insertion events into the ancestral chromosome 2L established *dm-W* as a female SDG. These three events lead to the generations of noncoding Ex1, Ex2-Ex3, and Ex4 of *dm-W* (this study). A less parsimonious possibility is that some or all of these three components were assembled in the diploid ancestor of subgenome S (or in subgenome S after allotetraploidization) and then translocated as a unit to subgenome L, with additional components being added there if the transferred gene was partial. The noncoding Ex1 were generated for promoter/enhancer for *dm-W* expression in gonadal somatic cells during sex determination. Ex1 may have arisen from an insertion DNA containing a repeated sequence, and then the promoter appeared to have evolved de novo. Ex2 and Ex3 evolved from the duplicate of the region covering Ex2, intron 3, and Ex3 of the S subgenome-derived ancestral *dmrt1.S*, which had been inserted into the ancestral chromosome 2L of the L subgenome probably through TEs (supplementary fig. S1, Supplementary material online) (Bewick et al. 2011; Mawaribuchi, Takahashi, et al. 2017). Ex4 was generated from the *hAT-10*-derived sequence, which had inserted downstream of the predicted insertion position of the partial duplicate of *dmrt1.S*. Our analyses were unable to determine which insertion occurred first, or whether they occurred concurrently. However, divergence times of Ex4 (fig. 2) and phylogenetic relationships among species that carry Ex2 and Ex3 (Cauret et al. 2020) suggest both were in place either around the time of allotetraploidization or relatively soon thereafter (fig. 2). *Xenopus borealis*, which is more diverged from *X. laevis* than the three other species examined in this study (*X. itombwensis*, *X. largeni*, and *X. petersii*) has independently evolved sex chromosomes (Furman and Evans 2016), and efforts to identify *dm-W* in this species were unsuccessful (Furman and Evans 2016; Cauret et al. 2020). We also did not detect the Ex4-like sequences in the whole genome sequence of *X. borealis* or isolate it by PCR. *dm-W* Ex2 and Ex3 were present in the ancestor of *X. clivii*, which is more closely related to *X. borealis* than to *X. laevis*, which suggests that Ex2, Ex3 and Ex4 of *dm-W* were lost in an ancestor of *X. borealis* after divergence from *X. clivii* (this study; Cauret et al. 2020). This loss may have been associated with the origin of new extant sex chromosomes in *X. borealis*, occurred independently in an ancestor with the same or different sex chromosomes, or the loss could have occurred after the new sex chromosomes of *X. borealis* evolved.

In conclusion, we found that the *hAT-10* transposon contributed to the birth of the chimeric SDG *dm-W* in the ancestor of the allotetraploid *Xenopus* frogs. This adds to a small number of examples where noncoding portions of TEs become CDS, and is the only known example to our knowledge of this process being a prelude to the de novo origin of an SDG. Recently, a retrotransposon-derived sequence was discovered to influence the sex-determining function of *Sry-T* as a splicing variant of *Sry* in the ancestor of mice (Miyawaki et al. 2020). However, this retrotransposon-derived sequence was not involved in the original emergence of *Sry* in the ancestor of therian mammals. *dm-W* is distinguished from this example because the *hAT-10* transposon-derived sequence was involved in *dm-W* birth in the ancestor of allotetraploid *Xenopus* frogs. In this way, this study provides new insights into de novo origin of SDGs.

## Materials and Methods

### Bioinformatic Analyses

Genomes analyzed in this study were obtained from Xenbase (www.xenbase.org/entry/; *X. laevis* v9.2 and *X. tropicalis* v10.0). Comparative analysis of the *dmrt1* subfamily was performed using mVISTA (http://genome.lbl.gov/vista) using the LAGAN alignment program (Frazer et al. 2004). Accession numbers of *X. laevis dmrt1.L*, *dmrt1.S*, *dm-W*, and *X. tropicalis dmrt1* used in the study are NM_001096500, NM_001085483, NM_001114842, and XM_031890717, respectively.

Phylogenetic analysis was performed using the software MEGAX (www.megasoftware.net/). Nucleotide sequences were aligned using MUSCLE (Edgar 2004) and gaps (insertions/deletions) were removed. Phylogenetic trees were constructed using neighbor-joining (NJ) and maximum likelihood (ML) methods. In NJ and ML analyses, the best-fit model of nucleotide substitution was selected by model selection using a likelihood ratio test. The Tamura three-parameter (T92) + G and Kimura 2-parater (K2) + G was selected for analysis of DM domain sequences (supplementary fig. S3, Supplementary Material online) and *hAT-10* sequences (fig. 3), respectively. Phylogenetic support for each node was assessed using nonparametric bootstrapping (Felsenstein 1983) with 1,000 replicates.

TEs were detected using CENSOR software (www.girinst.org/censor/index.php) (Kohany et al. 2006) and RepeatMasker (www.repeatmasker.org/) with default parameters and the *Xenopus* TE dataset obtained from Repbase (www.girinst.org/repbase/). DNA transposon *hAT-10*-derived sequences, including *Xt hAT-10* transposase-like, Ex4-like, and Ex4-CDS-like sequences (supplementary table S3, Supplementary Material online), were identified using TBLASTN (<1e−6 of *E*-value), BLASTN (<1e−6 of *E*-value, >65% sequence identity), and BLASTN (<1e−6 of *E*-value, >65% sequence identity over >60% of query length), respectively. The values were obtained after removing duplicate fragments of 10 kb (*Xt hAT-10* transposase-like), 1 kb (Ex4-like), and 150 bp (Ex4-CDS-like).

The repeat landscapes of the TEs were plotted using RepeatMasker and Python scripts (supplementary files S1–S3, Supplementary material online). Pairwise genetic distances were estimated between all sequences within each TE subfamily using MAFFT (Katoh et al. 2002), and phylogenies were constructed using FastTree (Price et al. 2010) based on the default Jukes-Cantor + CAT model. Consensus sequences of the TE subfamily were obtained from Repbase (Jurka et al. 2005) except for that of *Xl hAT-10*, which was determined by collecting *hAT-10*-like sequences among the existing transposons in the *X. laevis* genome sequence and constructing them using a simple majority rule based on a multiple alignment by BlastViewer. The age of TE families can be roughly estimated from the distances between each sequence and the consensus (Kapitonov and Jurkal 1996). The divergence time between the homologous regions of the most closely related *hAT-10* DNA transposon and *dm-W* Ex4 was estimated using a Jukes-Cantor-corrected substitution rate of 3.0 or 3.2 × 10^−9^ substitutions per year, which was calculated from synonymous substitution levels between *X. tropicalis* and *X. laevis* orthologs, and between *X. laevis* L and S homeologs, respectively (Session et al. 2016).

For contig assembly and scaffolding of the genome of *Xenopus borealis*, contigs were assembled using ABySS (http://github.com/bvgsc/abyss) under the conditions of (*k* = 83, *B* = 30G, *H* = 3, kc = 3, *v* = −v) and female or male whole genome sequence, SRR6357672 or SRR6357673, respectively. The two resulting assemblies were merged, and reference-guided scaffolding was performed with RaGOO (Alonge et al. 2019) -T sr using *X. laevis* v9.2 genome assembly as described (Suda et al. 2022).

### Isolation of *dm-W* Ex4 from Several *Xenopus* Species

Genomic DNA was purified from adult livers of three *Xenopus* species, *X. largeni* (MCZ-A cryogenic 333), *X. itombwensis* (MCZ-A A136197), and *X. petersii*, using the phenol–chloroform extraction method. PCR was performed using KOD FX DNA Polymerase (TOYOBO, Japan). A pair of primers, 5′-AGTTACATTACACCTCATCCTG-3′ and 5′-AGACGAGGAGTGTTATCCCTC-3′, were used for amplification of Ex4. The obtained DNA fragments were inserted into *Eco*RV site of pBluescript KS (+). DNA sequencing was performed using BigDye Terminator v3.1 Cycle Sequencing Kit (Applied Bioscience, Waltham, USA). Gene bank accession numbers for the Ex4 sequences from *X. largeni*, *X. itombwensis*, and *X. petersii* are LC699248/LC699249, LC699247, and LC699250, respectively.

### Construction of an Expression Plasmid for the C-terminal Truncated DM-W

The CDS for *dm-W* from 1 to 123 amino acids was amplified from cDNA by PCR using pcDNA3-FLAG-DM-W (Yoshimoto et al. 2008) as temperate and a pair of primers, 5′-GCTTATCGATACCGTCGAC-3′ and 5′-CTATGAAGTGGGTGTGCTG-3′. The amplified DNA fragment was inserted into pcDNA3-FLAG vector (Ito et al. 1999) in frame with FLAG-tag, resulting in the construction of pcDNA3-FLAG-DM-W Δ124–194.

### Electrophoretic Mobility Shift Assay

The two oligonucleotides, 5′-CCATCGAGCAACAATGTATCAAATCTC-3′ and 5′-GGGGAGATTTGATACATTGTTGCATCGATGG-3′, were annealed and labeled with 32P, using the Klenow fragment. Proteins were produced using the TNT Quick Coupled Transcription/Translation System (Promega), using pcDNA3-FLAG-DMRT1, pcDNA3-FLAG-DM-W (Yoshimoto et al. 2008), and pcDNA3-FLAG-DM-WΔ124–194. The resultant labeled DNA and each protein were mixed in a reaction buffer (10 mM Tris–HCl [pH 8.0], 100 mM KCl, 10% glycerol, 5 mM MgCl_2_ 0.075% Triton X-100, 1 mM dithiothreitol, 1% bovine serum albumin, 1 µg/µl poly (dI/dC), 1 mM spermidine) and incubated on ice for 30 min. The samples were subjected to electrophoresis through a nondenaturing 5% polyacrylamide gel containing Tris/glycine/EDTA (50 mM Tris, 380 mM glycine, 2 mM EDTA) and 2.5% glycerol in Tris/glycine/EDTA at room temperature. The dried gel was autoradiographed with a Fuji super RX film (Fujifilm) at −70°C.

### Western Blot Analysis

Immunoblotting of in vitro synthesized proteins was performed using an anti-FLAG antibody, M5 (Sigma), followed by an HRP-conjugated antimouse antibody (Sigma). The reaction was developed by enhanced chemiluminescent staining using SuperSignal West Femto Maximum Sensitivity Substrate (Pierce), and the signal intensity was measured using ImageJ (NCBI, MD).

### Luciferase Reporter Assay

HEK293T cells were cultured in DMEM containing 10% fetal calf serum. Twenty-four hours before transfection, cells were plated at 1 × 10^5^ cells per well in a 24-well plate. The cells were transfected with the firefly luciferase reporter plasmid p4xDMRT1-luc (100 ng) (Yoshimoto et al. 2010), effector plasmids, and *Renilla* luciferase vector pRL-SV40 (20 ng) (Promega) using TransIT^TM^-LT1 (Mirus). Total DNA was maintained at 500 ng per transfection with the pcDNA3-FLAG empty vector. After 24 h, activities from the two luciferases, which have dissimilar enzyme structures and substrate requirements, were measured in a Luminocounter 700 (NITI-ON) using the dual luciferase assay system (Promega). Firefly luciferase activity was normalized to *Renilla* luciferase activity.

## Supplementary Material

Supplementary data are available at Molecular Biology and Evolution online.

## Supplementary Material

msac138_Supplementary_Data
