# Supplementary material for: Neofunctionalization of a Noncoding Portion of a DNA Transposon in the Coding Region of the Chimerical Sex-Determining Gene dm-W in Xenopus Frogs
Source: Mol Biol Evol. 2022 Jun 28;39(7):msac138. doi: 10.1093/molbev/msac138 (PMC9250109; doi:10.1093/molbev/msac138)
Supplement: msac138_Supplementary_Data [file msac138_supplementary_data.zip › Supplement0528_final.docx]

**Supplementary information**

**Supplementary figures**

**Supplementary fig. 1. *Xenopus* frog diversification and the emergence of *dm-W* after allotetraploidization**

The figure was constructed based on finding from five studies (Evans et al. 2011, 2015, 2019; Session et al. 2016; Mawaribuchi et al. 2017b).

**Supplementary fig. 2. Patches of homology among four *Xenopus* *dmrt1* homologous genes, *X. laevis (Xl) dm-W*, *Xl dmrt1.L, Xl dmrt1.S,* and *X. tropicalis (Xt) dmrt1* using VISTA tool**

VISTA plots of the four genomic regions (100 kb of Scaffold78 on *Xl* chr2L, 96 kb on *Xl* chr1L, 75 kb on *Xl* chr1S, and 73 kb on *Xt* chr 1) were constructed by using *Xl dm-W* (upper panel) and *Xl dmrt1.S* (lower panel), respectively, as references. Dark blue and light blue indicate homologous coding and non-coding exons of genes, respectively. Pink shows conserved non-CDSs.

**Supplementary fig. 3. A phylogenetic tree of the regions containing the DM domain-coding sequences among *X. laevis* (*Xl*) *dm-W*, *dmrt1.S*, *dmrt1.L*, and *X. tropicalis* (*Xt*) *dmrt1***

The tree was constructed using the MEGAX program, based on about 1700 nucleotide sequences from the upstream region of Ex2 to the downstream region of Ex3 of *dm-W* and its corresponding ones of *Xl dmrt1.L and Xl dmrt1.S* and *Xt* *dmrt1* as an outgroup. In the NJ and ML analyses, the best-fit model of nucleotide substation was selected by model selection using likelihood ratio test. An identical topology was obtained for genes in both analyses. The ML tree is shown as a representative example. Numbers at each node denote the NJ/ML bootstrap percentage values based on 1000 replicates.

**Supplementary fig. 4. Density of transposable elements (TEs) in *X. laevis dm-W*, *dmrt1.L*, and *dmrt1.S* and flanking regions**

CENSOR program was used for the density of TEs, which shows a color gradient between blue (low) and red (high). Noncoding exons and coding portions of exons are represented by white and grey boxes, respectively. Numbers over each intron shown by horizontal black line indicate the proportions (%) of each intron that is comprised of TE sequence.

[exon 3] 5’-

**GTTGCATTGCAAAGACAGCAAGCTCAGGAAGAGGAGCTAGGAATATACCATCCTATTCCTTTGCCCATTGCAGCTGTGATAAAAAGGGAGCATGGTGGTAGCAGCTCTCAATTGATGCTGGAAAGCAGTTCCACACAGACAACCAGCACACCCACTTCAG**

[intron 3]

gtaacagaaggagcagggcagtgcaaataacatttaagatctagtgactttgtactgagaactggcaaaaagccaccctacccctttgcccattgcagtagagtagtggataaaaagggagcattgtggtaacaggccttgcttaatgcacagacagcagtcatacatactaccatctccacacccagctcatgtaaagatacagtatttttgctttgtagcaggtcaatcaagattgtattttggatctattttagtgaatcattttgctgatcacgcaccttaatttctgtatatgaaagaaacagcttgttctttgttcatcgggtatgcttttaggaagacttcttttcctacataaacaaaaacatatgtacaaccccttatggtattggcacacacagaatttattgatgtacagtatatgtgactctgccctgaaaagctaagtattatctttttacaaaaagacaattgctgttgtgttgagcacagtttgttccctaaggctagtgtcagatgtagcgggcattgccttggccagggttcagacacacatggtggatttcagggccaaaatgcaaacacttgcttttggcacagaaatgtgctgtgtgtgtctgaaccctggccaaggaaatgtatctgagtgcaggcacatcagcagaggagtggattctcagtttctctgcagactgagatccactacgtctggcactagccttagagccctgttacacgggcggcttcattgcctaatgggaaacctcctctcctcctcatctgcaccaggcagtgatcctaggtagttagcctgaaaatgcataaggaggcatttataagcaattacccaagatcgccacatgtaatgtgtaacacgtggtgattccaactaatgcgcatcagcaggtattttcagatgcttcatcacctgcagaacagattatttcccgctgggaaatgtagtgtcctgtgtgtcagcagccttgggatgctagaactataggcctgtgcaagcaggtggaagatgttgtgtgtaacacttacgttatcaggaaccttgcgcagtgacggataccctcgaatgcacaggtggaccctgtggattccggtgttcaccgacacccttttggggccccgggcattctgctgcagaaaccaacaaggatgtgcatagctagaaactagtccataagctaggtaccggcaaggagttatcaagttatcaaggcaggcagcaggaattgtagtcaagggtcaggcggaagtcaaaaaccaggagatcaaagtcaaaaaacactttagcaggaacagtaaactgaagcctagcttgggcactatcaaaatggtggatcaggcttttaaagaagaattggcgccaaaagattcaaaccggtgctgaatagtgacgtcatgatgctcagcatcggatttctaatgcggaggtgcgctcctgtgcacacgagcaagcatagaggtgtgccgggaggtaagcgaggagttagatctccctgcgtgtcttacagtgtggactaattaagagcacacgttttaagggccccttacacacaggcgttctgggctgctctccactgcattctgtctaaagccgtttgactgcaggggagcgcaggcccagacgcactctattcttacctatggtgctgtgacaatgcatgttgtgttccacctgtgtttggcacttacatgcgttgtcatgaagacagcaccataggtaagaattaagtgcatctgggcctgcgctcccctgcagtcaaaatgctttaaacagaatgcagtgcagtccagaatgcccgtgtgtaagggcccttagaatgctagtgataagttaactattggaaggagatctcatatcagataactaaaatggcaaactacataaagttgtacaaaacagcaggaggcctagaaagtctttcacggctggtgctaaaataatattaagggacaatagtagtaattggctttgccaagacactatgtgtgttttagtagagaccatgcccagtattgtgtatggcagggtattttttggcattttgtaggcaagtagcttctactagtagtgcagtgtatcttcaccctaaagggtgtacaaacaatgggagccctaaaaagatcacttatgagtgcaagtgcaaagtgttgaaatgtagtgatgggcaaataaatttgtcttagtgaaattcgcagtgaatttgagttttgccaggggcaaattttttttgcgaaactgcatcaaaaatttgcatgctactccacaacagcccagcccacataccatgcctagtctgggcactgtaatgcagcagctgcaatcacaaaaacagcagcagcaagcgccacagcagcagatggcattgggtcacagtggggcaatttttttgccactgcttcgcaacccatttgctcgcagcaggcagaggcagtggtagactggctgacccaggctaccttgtctgcagcgggcaccagtgagtggcaggaggtggcatcacctgcaagctcagtgctgggggaaaccccatcccccttattgtttgatcctgaggtggatttcggcccattcacccaggatctttttgatgatcaggcaggaatgggggtacattcatgtctgatgaggaggaggaggtcatggcacatcccacatcagggtcccctacagtggcggaactaccgggggagcagggggtgtgagcgggcgagggcccgcaccccctcagggcccccggcagtccattcgcctctgaaaatgcggccgtacggaggggggcggggcccggctgcgcatcacgcaccagggtccgcccccctctaggatcgctactggtcccctaatggcagggtagcaggctgctggtgtggggtcagacaccagcatgctggatgtgggtgtggatgacctactgcaggatctgttctgggggaggaggatgatagggacagaccctgggagctggctcaggaggataacatcagcagtttagggggggaactgtgtgttgttgttgaggatgatgatgaagagctggccccacagaggcagaaaaccccaccaacctttgctaggggggcaggcaacagggcagcactgcacctgggtccagagtctctgggcatatgggtggggaccattttcagccctccacagcaggcaaggcggtggcccacacttcagcagtgtgggcatttttcatgtgccagaatgaagaccagtcagtagcagtgtgccagctctgctagcagaaagtttgaagggggcagatggtcacatatgggaatgtctgctttaagttcccatataaaacatcatcataggatgatgtgggagcagcaccaaagtgccagggcactggggtgcagtggtgcttccggtccacctcctcccatttctcagggaagaggtgctagctccccatcccctgtagcaagggaagagtattctgaggctcactgcctcctcctcctcactgccatcatcagtgcagccccagcaggtggtggagaatgtgtccctgtcactggatcatgctgtcggcggcagagtgcacggatatgtggactagcaggcatgggcaggggaggtacatctcgtataccgcccactgggtcaccctcatgagtgctggggagggtgcaaggagggtgctcccctcaggctagaggtgcctccctgtggtgtacagggcaaacaccacatggtcacttcctcctccgctatggatgagccaccacttaagtgtccctgcagttacgcttcagtgcagcacaagcgcggtcaggctgtgctgcacctgagctcatggccgccttccagacttcacatgtcctcaaccttgtggtgcagcacttcttgaagagctaccaagggttgggtgacttggagaaggcacgtatgatatgcagccacttttgcaggtcccccaccgccagcacgtcttcggcatggatgcagcggcagcatcatctgccaccccactggctgatctgtgacctgacgatgcactgtaattccaccctgcacatggtggagtgccgtgtagagcagcgctgggtggtcagcaattagctgctggagcacaatgccaggggtactcacgggcagatttggggtacttcagtgccgagtagtggtagcagatgaggcagctctgctgagtacttgccccctttgagcaggccacacgctttgttagcagggacaatgcatgtgtgagtgatgttatactcctggttttcctcctcaatcgcatattgtacttctgctagaggagggcgatgtgcctgaggaggagaatagaccccataggcaggcagaaggggctgagaggcaggaggatgaggaagttttgtccgatgaggaggatgagggagaggaggactgggtgcctgcacagcatggggagcagggcacacaccatccaaccaccccagctattgtctgcggctgggagggcacagagcaggtggaggaggtagatgaccttctgcatctggagggtagtgaagaagaggttggtcgggggcacctcttttacatgtctgccaatatgcagagttgcctcaggagtaatcccccggatctgttccatcaaagaccgggatgattactgggtggccactttgcttgacccaccgtacaatggaaagttgggggagttccttgtgcccagccagagagaaaggagggcgggtcaattgaagagggctctgtgctcaaaactggtggaggccttcccccaggctgacacttctcaggcctccactaatccacacatgcagcagagatgggggcctagcagcagcagcaaaggcggagatctcatgggtgtgtggaagagcttttttgagcctcattaggcagcagcaggcccattcagcacccaaagttaccaccaacagcggctggagcatatggtggctaactacatggggtcggtcactgaagaccccatgcatttttgggtgttgaggctcaaccagtggccagaactggtacagtatgctctggaggtgcttgcttgcccccctgccagtgtcctgtcagagaatgtcttcaatgctgcaggtgggttggtcactgagaagcagacacggctgtccactggcagcgtggatatgctgacatttataaaaatgaatgaggcatggataagcgggatagccaagtgcctattgcagacattagagactagactcctctcctcttcctctccagatgtaccctcctctctccattgctgcctatactctcctctgatagacttctcctccatagttttgcctattccccatctggctgatatgtctgctcctgctgctgcagcctgctacccctagtactactgctacattgtctgcaatttttttgtgatgggggcctatcaatgctcctactactgtactgctgcatttttggcaaatgtttttgtggtggggtcctatcaaggctaataataatgttgcttctaatactgccacatggttggcaaatttcttctatttggggtctgtcaaggctcctaacttactaatactgctgctgcatagttgaggcctaccatggcttctaaaaatgttgcttctaatactgctgtgtagttggctaatgtcttctggttgaggcctacctcatctatttctaataatgttgattccagtgctgctgttgctactactgccacattgtagtcttatttcttctggttgaggcctaccaaggctaataataatttcacttttaatactgccgcattgttggctaattttttctggtggaggcctacctcttttaattctaattctaataatgttacttctaatactgcagcatggttggcaaatgttgtcacactattactaaaataaaaaaacataatgtatttgagtgttcctcaattcaatagctaattcatcaaattctgcccctgcacatcacacttggcacgtgagcatcaaactcggagccagcgtgtcaaaatcaatgtgtgtgcatcacgtgacacgcatgttatacgcacatcaaaaatgggtgtcactggtataagtacccaacgtccagctcttacacgtgtaggattggaaaacttttctacagcaaagatggcaggtccatgcctgatgagtgaagatcagataaattatttcatcagctacctgcagaggaagggatatgacaatatcccccctgtaacccccgggatacagtccctgcaacgtgcaataatggagaggctgaggcacaagctcaagaggcagtttcacatccgtctgaggctgcatgccatccaaagaatatggggcaatcttaagcggtggcagaccttgttaatcaactaagagctgaagtagaaggtagattaatttaaacagaataaatagatttctcttcttaaagggatcctgtcatccgagaacatgtttttttcataacacaccagttaatagtgctgctccagcagaattctgcactgaaatccatttctcaaaagagcaaacagatttttttatattcaattttgaaatctgacatgtcaatttcccagctgccccatgtcatgtgacttgtgcctgcactttaggagagaaatgatttctggcaggctgctgtttttccttctcaatgtaactgaatgtgtctcagtgggtttttactattgagtgttgttcttagatctaccaggcagctgttatcttgtgttagggagctgctatctggttaccttcccattgttcttttgtttggctgctggtggggaaaagggaggggggtgagatcactccaacttgcagtacagcagtaaagagtgattgaagtttatcagagtcacatggcttggggcagctgggaaattgacaatatgtctagccccgtgtcagatttcaaaattgaatataaaaaaatattttgggaaatggatttcagtgcagaattctgctggagcagcactattaactgattcatttcgagaaaatgttttttccccatgacagtatccctttaactacaataaagcaacttgattcctatttattttgctatttttgtgcaaatgtgtgtttgtttagacctgtaccaaggctggcgcatagttaacaaataaaggtagatttatttaaacagaataaatagatgtcctatactacaataaagccacttgattcctttttattttgctatttttgagcaaaagtgtgtttaggtgaatagtgtaacatccgtaacagttacatcatataactgcagagaacaagtaaatcaaatggagattatctgctgcaaaaaacctatcttaatggtgttgcctgccccatacccgccaatgccatccttataaatgtaggaggataaatgttttgctaaccttttggttctttgacattgccgccaggttagggactcaagtagttaagctcagggatagagagattcttggagtggaaaccagctgtgaggtcgccttcaatggggaatgtgcaccaaaggcaggaaaggcggctcaattcacaaagctgactaacaaaggatcagactgtatctgtgagtaatacatttgtgtaaatgacctgctgccactacttgttcatgaagtgtaacatctgagcattcatcttgaatatgtgagtattgagaagtgtaccgccaccaataaatgttctgctgttttaaagaaccactggcgctcaaagtttaattgatcaacagtgaatactgtgtgtgcttataccatgtggtaatcccttcctctataccataagcgagggccccacctgtggataaagaatctattgatatcgtgcctatcgggagttgctgggagttggaccccttgcccaggtaccgaaaccagtggatagtaacccaagggaaggtttaggaagaccctggcccatccacctgttacatagatgttgtttaaatttcctgaagttacattacacctcatcctgtgtaattcatactgtacaaatgcaattgctgtattactcaaactgaaattctttaattggcag

[exon 4]

**CTGAATGGATCAAGGAAGAAGAGGTGGCTAAACCTGCTGCAGGTCTTTTCGCACCTCCACCATCATCTGAAGAGATGGGCATGACACTGGCAGCAGTTGGAGCTGCTGATGCTGCAACACAGACTGATCAGCCAGGCCACCTCTTCAATATGATGTCTGCGATGATCCAGCAGCTCATGGACATGAGCTGGGAACTCCAACAAGGATGGATTTAA**TTAATAGTTTGAGGGATAACACTCCTCGTCTAGTAGTCTTTTAGTTGTATGCACTATTGTGCCCTATGTTGTATGCACTGATGTGCCTTATCTTTTTTTTTCTATAGTAAAAAATCTGTTCAAACCTGTA

[3’-flanking sequence of exon 4]

tatttggagtttttttttttcatttaactactactctgataatacagaagttgtttggataatatgacacaactaggactattctcggtcatgtaacattacattacctagtatttctgattccttacatttacacataaatatatggtttattcattttaatgtgggatacaaaacacctacatatgtgttaagatttattattggcaagctggcaaaatgggagcacttttttttcttttgaaaagaaggagcaggcgcagaagcggtgatgtcagcgtctggccccagggcacgcttgcgtgatgcgtcattaggcacacaggagggattatcccagtggttgttatttctgttagagagatagatagataatagatagatagatagatagatatggatgatcaagtgccaagtagcccagacaatgctgcaaatgaaaataggaggtgagccacggtcacgcagggccggaactgggggtaggcagagtaggcggctgcctagggcgccaatcctgaggggcgcacttttaaggggtttttttttttttggttttgcttatttaataatttatttaagaaatcatggtcgggcaaagtccattttatccccggcctcacctccctcttgccggcaattgatagctccctcctgcctccttctgtgcggtgcgatgtgtcggctcgtcgacgcaatgtgcataagtcagtcaatgatgtcactaacgtggctggtcacaggagagaggctgagaactgacaggcctaggcgcgtttgtggagtttgtgctcgctgctctgtctcagccttgcatgcacagttcagcctgaacttctattactgttacgcttcctgctggcacagccatcccaggtacagatttgagggccttatttttgctattgttgctgggcactggcacaggtttaaaaatactgggggcaatatgatgtgtaaagattggggacaatatgctggctgctgggcttgtgtgccagcagtacaggagggctgtatgattggtggctgctgggtttgctggcacaggtatgacttggggggctgtatgatggatggctgctgggcatgctggcataggtatggcaggctgtatgatgggtggctgtcgggcatgctggcacaggtaggggggttgtatgatggatggctgctggcacaggggggggggggcgctggaagaagctcttgcctagggtgccaaactaccttggcccggccctgctgtcacggtaagtttatagtgttggtaaaaaaaagagtgctaagataattttgttcttttctcagtgtgtcttcccctagaaagaggaccagcaaggagaaaaataagaattatgttgcatgtgacagtccagcaatgaagcattcaaggttgtgtcaaagatgcacaagaaggctgttgggtgacgcagcagcagatacagtggatgccat -3’

**Supplementary fig. 5. Nucleotide sequences of *dm-W* Ex3, Int3, and Ex4**

Black or white upper letters indicate coding sequences (CDSs) or non-CDS of *dm-W* exons, respectively. By contrast, lower letters show non-exon (intron or 3’-flanking) sequences. Shading indicates other features of interest, as follows: dark green, transposase coding-derived sequence of *hAT-10*; bright green, *hAT-10* sequence except for its transposase CDS; black, 14 base of terminal inverted repeat; light blue, other DNA transposons than *hAT-10*; purple, retrotransposon. Splicing donor and acceptor sites in intron 3 are underlined.

A　　　　

B

**Supplementary fig. 6. Nucleotide sequence alignments among five *hAT-10-*derived sequences including *dm-W* Ex4 from *X. laevis* and its corresponding one of *Xt hAT-10***

(A) Sequence alignments of the regions A, and a part of B (see Fig. 1) among the five *X. laevis hAT-10*-derived sequences on *dm-W*-containing scaffold 78, chromosome 2L, chromosome 7L, scaffold 19, and scaffold 30, and *X. tropicalis hAT-10.* Identical nucleotides among all the five sequences and four sequences are shown by white letters on black background and black letters on grey background, respectively. The numbers on the alignments indicate those corresponding to *Xt hAT-10* sequence. Pale blue, pink and grey horizontal lines indicate the homologous region with *Xt hAT-10* sequence, *Xl dm-W* Ex4 CDS, and its non-CDS, respectively.

(B) Sequence alignments of the regions A, B, and a part of C (see Fig. 1) among the three *hAT-10*-derived sequences on *dm-W*-containing scaffold 78, scaffold30 and *Xt hAT-10*. Pale blue, pink and grey show the same as in (A). Nucleotide sequences in pale blue-violet shading indicate the region which was recognized as *Xt hAT-10* sequence by CENSOR program. Asterisks represent identical nucleotides among the three.

**Supplementary fig. 7. Sequence comparison of *Xenopus* *hAT-10*-derived A-C regions**

(A) Schematic comparison among *Xenopus* (*X*) *tropicalis* and *X. laevis hAT-10*-derived A-C consensus sequences and their surrounding regions. The numbers under the A, B, and C regions show nucleotide identity (%) in pairwise comparisons between genes depicted above and below the values. (B) Sequence comparisons of the eight *X. laevis* and six *X. borealis* *hAT-10*-derived A-C sequences in Figure 2A and supplementary table 2, respectively, which were constructed by Jalview (<https://www.jalview.org/>). Nucleotides A, T, G, and C correspond to light green, blue, red, and orange, respectively.

**Supplementary table 1. Nucleotide sequence identity (%) of three regions A - C for comparisons between *dm-W* and *X. laevis* *hAT-10*-like sequences**

|  |  |  |  | |  |  |  |  |  |
| --- | --- | --- | --- | --- | --- | --- | --- | --- | --- |
|  | **A. Sequence identity of *dm-W* Ex4-containing sequences to *Xl* and *Xt* *hAT-10-*like sequences** | | | | | | |  |  |
|  | Search global homology | | |  |  |  |  |  |  |
|  |  |  |  | |  |  |  |  |  |
|  | Region | scaffold78 (*dm-W*) | chr2L | | chr7L | scaffold19 | scaffold30 | *Xt hAT-10* |  |
|  | A | 149 bp | 79.8% (119/149) | | 83.0% (118/142) | 77.1% (115/149) | 88.3% (129/146) | 73.4% (108/147) |  |
|  | B | 219 bp | 85.4% (182/213) | | 77.1% (169/219) | 72.6% (157/216) | 74.0% (160/216) | 43.3% (95/219) |  |
|  | C | 315 bp | 48.8% (148/303) | | 53.9% (137/254) | 58.0% (97/167) | 79.8% (241/302) | 71.5% (189/264) |  |
|  |  |  |  | |  |  |  |  |  |
|  | Muscle alignment | |  | |  |  |  |  |  |
|  |  |  |  | |  |  |  |  |  |
|  | Region | scaffold78 (*dm-W*) | chr2L | | chr7L | scaffold19 | scaffold30 | *Xt hAT-10* |  |
|  | A | 149 bp | 87.8% (129/147) | | 92.3% (120/130) | 85.7% (126/147) | 90.5% (133/147) | 81.8% (117/143) |  |
|  | B | 219 bp | 85.4% (182/213) | | 80.1% (173/216) | 74.5% (161/216) | 77.0% (164/213) | 56.9% (119/209) |  |
|  | C | 315 bp | 56.7% (161/284) | | 58.8% (147/250) | 54.9% (150/273) | 80.2% (243/303) | 76.4% (197/258) |  |
|  |  |  |  | |  |  |  |  |  |

|  |  |  |  |  |  |  |  |  |
| --- | --- | --- | --- | --- | --- | --- | --- | --- |
|  | **B. Sequence identity of *Xt hAT-10* to *Xl* *hAT-10-*like sequences** | | | | | |  |  |
|  |  |  |  |  |  |  |  |  |
|  | Search global homology | |  |  |  |  |  |  |
|  |  |  |  |  |  |  |  |  |
|  | Region | *Xt hAT-10* | chr2L | chr7L | scaffold19 | scaffold30 | scaffold78 |  |
|  | A | 179 bp | 77.0% (138/179) | 76.1% (131/172) | 75.9% (136/179) | 79.3% (142/179) | 73.4% (108/147) |  |
|  | B | 310 bp | 50.9% (110/216) | 48.9% (120/245) | 47.7% (125/262) | 48.9% (121/247) | 43.4% (95/219) |  |
|  | C | 280 bp | 49.2% (132/268) | 52.6% (130/247) | 47.6% (72/151) | 60.8% (166/273) | 71.5% (189/264) |  |
|  |  |  |  |  |  |  |  |  |
|  | MUSCLE alignment | |  |  |  |  |  |  |
|  |  |  |  |  |  |  |  |  |
|  | Region | *Xt hAT-10* | chr2L | chr7L | scaffold19 | scaffold30 | scaffold78 |  |
|  | A | 179 bp | 77.7% (139/179) | 81.9% (136/166) | 77.1% (138/179) | 80.4% (144/179) | 81.8% (117/143) |  |
|  | B | 310 bp | 66.5% (131/197) | 63.4% (149/235) | 60.9% (145/238) | 69.0% (149/216) | 57.2% (119/208) |  |
|  | C | 280 bp | 59.2% (154/260) | 53.8% (133/247) | 55.3% (140/253) | 63.8% (173/271) | 74.9% (197/263) |  |
|  |  |  |  |  |  |  |  |  |

Note: Sequence identities were examined by “Search Global Homology” in GENETYX-MAC or “MUSCLE alignment program”. More than 70 % identity between two sequences is shown in red.

**Supplementary table 2. Nucleotide sequence identity (%) of three regions A - C for comparisons between *dm-W* and *X. borealis* *hAT-10*-like sequences**

|  |  |  |  |  |  |
| --- | --- | --- | --- | --- | --- |
|  |  | Region | | |  |
|  |  | A | B | C |  |
|  | ***X. laevis*** Scaffold78 (*dm-W*) | 149 bp | 219 bp | 315 bp |  |
|  |  |  |  |  |  |
|  | ***X. laevis*** Scaffold30 | 88.3% (129/146) | 73.4% (163/222) | 86.4% (210/243) |  |
|  | ***X. laevis*** Scaffold19 | 81.4% (110/135) | 72.4% (163/225) | 77.0% (124/161) |  |
|  |  |  |  |  |  |
|  | ***X. borealis*** M Scaffold19005437 | 86.3% (127/147) | 73.3% (149/203) | 84.0% (238/283) |  |
|  | ***X. borealis*** M Scaffold18565119 | 82.6% (119/144) | 72.4% (150/207) | 83.4% (217/260) |  |
|  | ***X. borealis*** F Scaffold26834261 | 84.2% (118/140) | 76.3% (152/199) | 78.0% (227/291) |  |
|  | ***X. borealis*** F Scaffold26740558 | 80.1% (109/136) | 74.8% (152/203) | 83.8% (244/291) |  |
|  | ***X. borealis*** F Scaffold26616759 | 82.6% (119/144) | 72.4% (150/207) | 79.7% (229/287) |  |
|  | ***X. borealis*** F Scaffold12410863 | 89.7% (70/78) | 71.4% (148/207) | 75.4% (187/248) |  |
|  |  |  |  |  |  |
|  | ***X. tropicalis*** *hAT-10* (8560-9323) | 72.7% (107/147) | 43.1% (97/225) | 68.3% (177/259) |  |
|  |  |  |  |  |  |

**Supplementary table 3. GenBank assembly accession of 11 vertebrate species used in fig. 2.**

| *Rana catesbeiana* | RCv2.1 | GCA_002284835.2 |
| --- | --- | --- |
| *Nanorana parkeri* | ASM93562v1 | GCA_000935625.1 |
| *Rhinella marina* | RM170330 | GCA_900303285.1 |
| *Ambystoma mexicanum* | AmbMex60DD | GCA_002915635.3 |
| *Geotrypetes seraphini* | aGeoSer1.2 | GCA_902459505.2 |
| *Homo sapience* | GRCh38.p13 | GCA_000001405.28 |
| *Mus musculus* | GRCm39 | GCA_000001635.9 |
| *Gallus gallus* | bGalGal1.mat.broiler.GRCg7b | GCA_016699485.1 |
| *Oryzias latipes* | ASM223467v1 | GCA_002234675.1 |
| *Tetraodon nigroviridis* | ASM18073v1 | GCA_000180735.1 |
| *Callorhinchus milii* | Callorhinchus_milii-6.1.3 | GCA_000165045.2 |
