# Supplementary material for: Neofunctionalization of a Noncoding Portion of a DNA Transposon in the Coding Region of the Chimerical Sex-Determining Gene dm-W in Xenopus Frogs
Source: Mol Biol Evol. 2022 Jun 28;39(7):msac138. doi: 10.1093/molbev/msac138 (PMC9250109; doi:10.1093/molbev/msac138)
Supplement: msac138_Supplementary_Data [file msac138_supplementary_data.zip › Supplemental_file_1.pdf]

```
# Supplemental file 1

# Script Name: RMalign2divbed.py

#!/usr/bin/python
# -*- coding: utf-8 -*-
"""
    Usage: ./RMalign2divbed.py <*.align> <*.bed>

    This python3 script reads a single *.align file which is
    the output result of RepeatMasker and creates one BED files.

    Estimate pairwise genetic distances between all aequences within each TE subfamily
    using MAFFT (Misawa et al. 2002), and FastTree (Dehal et al. 2010) based
    on Jukes-Cantor + CAT model (defalt).

"""
#
# Module imports
#
import sys
import pandas as pd
import subprocess
import csv
import itertools
import re

args = sys.argv
RMal = str(args[1])
output = str(args[2])

# Divide repeat fragments
with open(RMal) as t:
        s = t.read().split(")\n\n")

# Run MAFFT, followed by FastTree
TE_count = len(s)
n = 0
with open(output, 'w') as o:
        for i in s:
                n += 1
                if not i:
                        pass
                else:
                        i2 = i.strip("\n")
                        i2 = i2.rstrip(")")
                        lines = i2.split("\n")
           # If the result is Simple_repeat
                        if "Kimura" not in lines[-3]:
               # Extract alignment data for each reference (ref) and TE library (lib) sequences
               seq = lines[2:-4]
                                seql = seq[2::4]
                                seqr = seq[0::4]
                                lib = ""
                                ref = ""
                                for r in seqr:
                                        r = r.lstrip("C")
                                        r2 = ' '.join(r.split())
                                        r3 = r2.split()
                                        ref += str(r3[2])
                                ref = ref.replace('-','')
                                for l in seql:
                                        l = l.lstrip("C")
                                        l2 = ' '.join(l.split())
                                        l3 = l2.split()
                                        lib += str(l3[2])
                                lib = lib.replace('-','')
                                aln = ">ref" + "\n" + ref + "\n" +  ">lib" + "\n" + lib
                                with open('aln_temp.fa','w') as g:
                                        g.write(aln)
               # Run MAFFT
                                mafft_cmd = ('mafft --retree 2 --reorder aln_temp.fa > aln.temp')
                                subprocess.run(mafft_cmd, shell=True)
               # Run FastTree
               cmd = ('fasttree -nt aln.temp > tree.temp')
                                subprocess.run(cmd, shell=True)
               # Export to an output file in BED format
                                with open('tree.temp', 'r', encoding='utf-8') as t:
                                        a = t.read()
                                        b = re.split('[:,]',a)
                                sub_rate = (b[1])
                                stats = lines[0]
                                stats = stats.split()
                                if stats[8] == "C":
                                        o.write(stats[4] + "\t" + stats[5] + "\t" + stats[6] + "\t" + stats[9] + "#" + sub_rate + "#" + "-" + "\n")
                                else:
                                        o.write(stats[4] + "\t" + stats[5] + "\t" + stats[6] + "\t" + stats[8] + "#" + sub_rate + "#" + "+" + "\n")
                                print(str(n) + " / " + str(TE_count))
           # In the case of something other than Simple_repeat
                        else:
               # Extract alignment data for each reference (ref) and TE library (lib) sequences
                                seq = lines[2:-5]
                                seql = seq[2::4]
                                seqr = seq[0::4]
                                lib = ""
                                ref = ""
                                for r in seqr:
                                        r = r.lstrip("C")
                                        r2 = ' '.join(r.split())
                                        r3 = r2.split()
                                        ref += str(r3[2])
                                ref = ref.replace('-','')
                                for l in seql:
                                        l = l.lstrip("C")
                                        l2 = ' '.join(l.split())
                                        l3 = l2.split()
                                        lib += str(l3[2])
                                lib = lib.replace('-','')
                                aln = ">ref" + "\n" + ref + "\n" +  ">lib" + "\n" + lib
                                with open('aln_temp.fa','w') as g:
                                        g.write(aln)
                                # Run MAFFT
                                mafft_cmd = ('mafft --retree 2 --reorder aln_temp.fa > aln.temp')
                                subprocess.run(mafft_cmd, shell=True)
               # Run FastTree
                                cmd = ('fasttree -nt aln.temp > tree.temp')
               # Export to an output file in BED format
                                subprocess.run(cmd, shell=True)
                                with open('tree.temp', 'r', encoding='utf-8') as t:
                                        a = t.read()
                                        b = re.split('[:,]',a)
                                sub_rate = (b[1])
                                stats = lines[0]
                                stats = stats.split()
                                if stats[8] == "C":
                                        o.write(stats[4] + "\t" + stats[5] + "\t" + stats[6] + "\t" + stats[9] + "#" + sub_rate + "#" + "-" + "\n")
                                else:
                                        o.write(stats[4] + "\t" + stats[5] + "\t" + stats[6] + "\t" + stats[8] + "#" + sub_rate + "#" + "+" + "\n")
                                print(str(n) + " / " + str(TE_count))
cmd2 = ('rm *temp')
subprocess.run(cmd2, shell=True)
```
