# Supplementary material for: Neofunctionalization of a Noncoding Portion of a DNA Transposon in the Coding Region of the Chimerical Sex-Determining Gene dm-W in Xenopus Frogs
Source: Mol Biol Evol. 2022 Jun 28;39(7):msac138. doi: 10.1093/molbev/msac138 (PMC9250109; doi:10.1093/molbev/msac138)
Supplement: msac138_Supplementary_Data [file msac138_supplementary_data.zip › Supplemental_file_2.pdf]

```
# Supplemental file 2

# Script Name: fix_TE_location.py

#!/usr/bin/python
# -*- coding: utf-8 -*-
"""
    Usage: ./fix_TE_location.py <*2.bed>

    This python3 script reads a single *2.bed file which is
    the output after the execution command show bellow:

    $ ./bedtools cat <*1.bed> | ./bedtools sort |
    ./bedtools merge -c 4,2,3 -o collapse > <*2.bed>

    Note: The *1.bed file is a result of the RMalign2divbed.py

    Resolve overlapping annotation. There are cases where
    RepeatMasker cannot automatically determine which is the
    best call and does print out overlapping annotations.

"""
#
# Module imports
#
import csv
import sys
import re

csvin = csv.reader(sys.stdin, delimiter="\t")
for row in csvin:
        TE_type = row[3].split(",")
        TE_start = row[4].split(",")
        TE_end = row[5].split(",")
        if len(TE_type) == 1:
                print(row[0],row[1],row[2],row[3], sep="\t")
    # List overlapping annotation
        else:
                lists = []
                for t, s, e in zip(TE_type,TE_start,TE_end):
                        t2 = t.split("#")
                        if t2[1] == "Simple_repeat":
                                lists.append([t,s,e,100])
                        else:
                                lists.append([t,s,e,float(t2[2])])
        # Sort lists by substitution rate
                lists = sorted(lists, key=lambda x:x[3],)
        # Remove overlapping annotation
                pre = []
                for l in lists:
                        if not pre:
                                pre.append([l[0],l[1],l[2]])
                        else:
                                for p in pre:
                                        if int(p[1]) <= int(l[1]) and int(l[2]) <= int(p[2]):
                                                l[1] = 0
                                                l[2] = 0
                                        elif int(p[1]) <= int(l[1]) and int(l[1]) <= int(p[2]):
                                                l[1] = int(p[2]) + 1
                                        elif int(p[1]) <= int(l[2]) and int(l[2]) <= int(p[2]):
                                                l[2] = int(p[1]) - 1
                                        else:
                                                pass
                                if l[1] == 0:
                                        pass
                                else:
                                        pre.append([l[0],l[1],l[2]])
                for p in pre:
                        print(row[0],p[1],p[2],p[0], sep="\t")
```
