# Supplementary material for: Neofunctionalization of a Noncoding Portion of a DNA Transposon in the Coding Region of the Chimerical Sex-Determining Gene dm-W in Xenopus Frogs
Source: Mol Biol Evol. 2022 Jun 28;39(7):msac138. doi: 10.1093/molbev/msac138 (PMC9250109; doi:10.1093/molbev/msac138)
Supplement: msac138_Supplementary_Data [file msac138_supplementary_data.zip › Supplemental_file_3.pdf]

```
# Supplemental File 3

# Script Name: TE_bp_caluculation.py

#!/usr/bin/python
# -*- coding: utf-8 -*-
"""
    Usage: ./TE_bp_caluculation.py <*.bed> <*.tsv> Arg[3] Arg[4] Arg[5] Arg[6]

    This python3 script reads a single *.bed file which is
    the output result of fix_TE_location.py and creates one TSV files.

    Contents of the BED file :
    chr         start           end             subfamily#family#time
    chr7L        13276152 13276251 Gypsy-7_XL-LTR#LTR/Gypsy#0.032127701#+

    Arg[3]  :  Classification of class of TE to output. (0: subfamily, 1: family)
    Arg[4]  :  Number of units of replacement rate to be extracted staring from 0.
    Arg[5]  :  The smallest unit of substitution rate to extract.
    Arg[6]  :  Decimal point indicated by Arg[5].

    Calculate the total length for each substitution rate in each TE subfamily or family.
    Open the output file in Excel and create bar graph as a repeat landscape.

"""
#
# Module imports
#
import sys
import csv

args = sys.argv
TE_bed = str(args[1])
output = str(args[2])
calc_class = int(args[3])
scale_count = int(args[4])
scale = float(args[5])
float_count = int(args[6])

with open(TE_bed) as t:
    TE = t.readlines()
TE_list = []
TE_loci = {}

n = 1
for t in TE:
    t = t.split()
    TE_stats = t[3].split("#")
    if calc_class == 1:
        TE_loci_key = t[0] + ":" + str(int(t[2]) - int(t[1]) + 1) + ":" + TE_stats[calc_class] + ":" + str(round(float(TE_stats[2]),float_count)) + ":" + str(n)
        TE_loci[TE_loci_key] = str(TE_stats[calc_class])
        TE_list.append(str(TE_stats[calc_class]))
    else:
        if TE_stats[1] == "Simple_repeat":
            TE_loci_key = t[0] + ":" + str(int(t[2]) - int(t[1]) + 1) + ":" + TE_stats[1] + ":" + str(round(float(TE_stats[2]),float_count)) + ":" + str(n)
            TE_loci[TE_loci_key] = str(TE_stats[1])
            TE_list.append(str(TE_stats[1]))
        else:
            TE_loci_key = t[0] + ":" + str(int(t[2]) - int(t[1]) + 1) + ":" + TE_stats[calc_class] + ":" + str(round(float(TE_stats[2]),float_count)) + ":" + str(n)
            TE_loci[TE_loci_key] = str(TE_stats[calc_class])
            TE_list.append(str(TE_stats[calc_class]))
    n += 1

TE_list = sorted(list(set(TE_list)))

lists = []
time= ["time"]
lists.append(time)
for c in range(scale_count):
    count = [float(c*scale)]
    lists.append(count)

for tl in TE_list:
    key = [k for k,v in TE_loci.items() if v == tl]
    temp_dict = {}
    for k in key:
        k = k.split(":")
        temp_key = str(k[1]) + ":" + str(k[4])
        temp_dict[temp_key] = float(k[3])

    temp_sum = []
    temp_sum.append(tl)
    for c in range(scale_count):
        count = round(float(c*scale),float_count)
        bp = [k for k,v in temp_dict.items() if v == count]
        if not bp:
            bp_sum = 0
        else:
            temp = []
            for b in bp:
                b = b.split(":")
                temp.append(int(b[0]))
            bp_sum = sum(temp)
        temp_sum.append(int(bp_sum))
    n = 0
    for row in lists:
        row.append(temp_sum[n])
        n += 1

with open(output,'w') as g:
    writer = csv.writer(g,delimiter="\t")
    writer.writerows(lists)
```
